# Dietary patterns and quality in West-African immigrants in Madrid

**DOI:** 10.1186/1475-2891-8-3

**Published:** 2009-01-23

**Authors:** Hélène F Delisle, Jesús Vioque, Augusta Gil

**Affiliations:** 1 WHO Collaborating Centre on Nutrition Changes and Development, Department of Nutrition, Faculty of Medicine, Université de Montréal, PO Box 6128, Downtown Station, Montreal, Que, H3C 3J7, Canada; 2 Departamento de Salud Pública, Facultad de Medicina, Campus de San Juan, Universitas Miguel Hernández, 03550 San Juan de Alicante, Spain; 3 CIBER de Epidemiologia y Salud Pública (CIBERESP), Barcelona, Spain; 4 Agencia Española de Seguridad Alimentaria y Nutrición, C/ Alcalá 56, Despacho 451, 28014, Madrid, Spain

## Abstract

**Background:**

Eating patterns of immigrants deserve to be better documented because they may reflect the extent of acculturation and associated health risks. The study assessed dietary patterns and quality in Bubi immigrants (from Equatorial Guinea) using cluster analysis and comparing different diet quality indexes.

**Methods:**

A random sample of 83 Bubi men and 130 women living in Madrid were studied. A 99-item food frequency questionnaire was administered, body weights and heights were self-reported and socio-demographic and health information was collected during interviews. Usual intakes were collapsed into 19 food groups. Cluster analysis of standardized food intakes per 1000 kcalories was performed. Dietary quality was appraised using the Alternative Mediterranean Diet Score, the Alternative Healthy Eating Index and scores of micronutrient adequacy and prevention based on WHO/FAO recommendations.

**Results:**

Two dietary patterns were identified. The 'Healthier' pattern, so confirmed by two dietary quality indexes, featured a higher consumption of fish, fruits, vegetables, legumes, dairy products and bread while the 'Western' pattern included more processed meat, animal fat, and sweetened foods and drinks. One third of the subjects were in the 'Healthier' food cluster, with the same proportion of men and women. Age ≥ 30 and residence in Madrid ≥ 11 years were independently associated with the healthier diet. Consumption of traditional foods was unrelated to dietary pattern, however. Overall, Bubi diets were somewhat protective because of high intakes of fruits and vegetables and monounsaturated fat (olive oil), but not with respect to sugar, cholesterol, omega-3 fatty acids and fibre. Less than two thirds of subjects had adequate intakes of iron, calcium and folate in both dietary phenotypes. Body mass index, physical exercise, and self-reported health and cardiovascular disease condition showed no significant association with the dietary pattern.

**Conclusion:**

Cluster analysis combined with dietary quality assessment facilitates the interpretation of dietary patterns, but choosing the appropriate quality indexes is a problem. A small number of such indexes should be standardized and validated for international use. In the group studied, younger subjects and more recent immigrants were more likely to have a 'Western' pattern and should be a priority target for nutrition communication.

## Background

There are several good reasons to study dietary patterns and quality, including the fact that interactions and synergistic effects of foods and nutrients on health are better captured by studying the whole diet than individual components. [1] Dietary patterns have been empirically derived using multivariate designs such as factor or cluster analysis [1-3]. The interest of dietary pattern analysis is that it is data driven and therefore allows for the complexity of dietary exposure [4], although the arbitrary grouping of foods and the often subjective selection of the statistical solution may be regarded as shortcomings. Cluster analysis is useful for identifying mutually exclusive groups of people with homogeneous dietary patterns, which is not the case with factor analysis. Health-related diet quality has been assessed using a variety of predefined scores or indices [5,6] that rate the diet in one single figure. While dietary pattern analysis is data driven, dietary quality indexes are based on *a priori *criteria.

The immigrant population is ever growing in developed countries. The assessment of dietary patterns and quality is particularly relevant among these groups as a means of appraising food acculturation and potential health consequences. Identifying the dietary patterns with the best health and nutrition profile bears significance for health promotion. Some studies suggest that recent immigrants are in better health than the host country residents – the "healthy immigrant effect" -, partly owing to health and wealth selection, but that with time, the prevalence of chronic disease factors among them reaches and even exceeds that of the native-born population [7-11]. Lifestyle has been incriminated, including dietary acculturation [12,13]. In the United States, it has been referred to as "unhealthy assimilation" [14]. However, there is no consistent relationship between duration of residence in host countries and mortality or morbidity and confounding factors include socioeconomic position, place of birth and ethnic group social network [15]. Immigrants from poor areas of developing countries may be at further health risk because of suboptimal nutrition in their early life, according to the theory of developmental origins of chronic disease [16].

The purpose of this paper is to characterize in Bubi immigrants in Spain dietary patterns using cluster analysis, dietary quality with a few indexes, and associated demographic and health-related factors. A published descriptive report on their diet is available [17].

The Bubi people are indigenous to Bioko Island, Equatorial Guinea. Of an estimated 85 000 ethnic Bubis remaining in the world, 45 000 live on Bioko Island and 35 000 in Spain. In the course of the 20^th ^century, the traditional Bubi diet based on roots and tubers progressively incorporated rice, salted-dried fish and other processed foods (eg. sugar, canned foods). Traditionally, the consumption of protein and fat from animal sources was very low [18].

## Methods

A random sample of 300 subjects aged at least 18 years was selected in the Bubi community of Madrid which includes approximately 3500 adult members. A total of 213 persons participated in the study conducted in 2001. The study was approved by the Board of the Bubi Association in Madrid. Each participant signed an informed consent form before enrolment in the study.

A food-frequency questionnaire (FFQ) with 99-food items was administered by trained Bubi interviewers to appraise the usual food and nutrient intake over the previous year. The FFQ was a modified version of the Harvard questionnaire [19] that was adapted for use in adult Spanish populations and validated against four one-week dietary records collected over a year among 106 adult men and women. Pearson's correlation coefficients after adjustment for energy intake ranged from 0.22 for monounsaturated fat to 0.56 for calcium intake. The average correlation for all nutrients was 0.38. Correlation coefficients between two FFQ measurements one year apart (reproducibility) showed slightly better results. [20-22]. For the purpose of the study among Bubi adults, six commonly consumed traditional food items available in Madrid (yam, cocoyam, cassava, taro, plantain and okra) and average portion sizes were added to the initial 93-item FFQ, following a pilot study among 30 Bubi subjects. Participants in the study were asked how often they usually consumed each item. The nine possible answers ranged from "never or less than once a month" to "six or more times per day". Combined with standard serving sizes, the intake frequencies were converted into average daily intake for each food item and for each individual participant.

Additional information collected during the interview and used in the present paper pertains to age, duration of residence in Spain, self-reported weight, height, health and health problems, and exercise and smoking habits. Further details of the study are available elsewhere [17].

Consumption of 19 food groups was computed and total energy and nutrient intakes were estimated using the comprehensive food composition tables of the US Department of Agriculture , which include data on practically all food items available in Spain and on a wide variety of ethnic foods.

Dietary patterns were identified using cluster analysis (Kmeans approach). Factor and cluster analyses are the main data-driven methods to study dietary patterns. Cluster analysis identifies mutually exclusive groups of individuals, unlike factor analysis which reduces dietary data into patterns based on correlation between foods [1]. Cluster analysis was selected because the results are easier to interpret and are better suited to targeting nutrition promotion efforts. Cluster solutions may vary depending on strategies used [23]. As advocated [1], we performed the cluster analysis on standardized daily intakes (z-scores) of food groups in grams per 1000 kcalories. This correction for energy intake prevents big eaters from exerting undue influence on the resulting patterns. Micronutrient intakes were also computed per 1000 kcalories to compare the micronutrient density of the dietary patterns. To assess dietary quality, we used the Alternative Healthy Eating Index (AHEI) [24], the Alternative Mediterranean Diet Score (AMDS) [25], and a prevention score and a micronutrient score based on FAO/WHO recommendations [26,27] that we developed and used in different population groups [28-30]. The latter two scores dichotomize intakes of 18 macronutrients, micronutrients and foods, giving one point if intake meets the recommendation and 0 if it does not. We used the AMDS because the study population lives in Spain and this index appeared particularly appropriate. Several randomized trials have shown the positive association of Mediterranean diets with the chronic disease risk reduction [31]. We also used the AHEI which is widely utilized and reportedly correlated with the AMDS and with biomarkers of cardiovascular risk [25,32,33]. Additionally, we tested our prevention and micronutrient scores because of their international scope.

The data were analyzed with SPSS 15.0.1 for Windows (2005). Food and dietary intakes were compared between food pattern clusters using Student t test. Chi^2 ^tests were used for categorical data on characteristics of subjects. The associations among dietary quality indexes were tested with Pearson's correlation coefficients. In order to explain the relationships between food patterns and demographic and health-related variables, we used logistic regression analyses. The statistical significance threshold was p < 0.05.

## Results

The study population consisted of 83 men (mean age: 33.2 y ± 12.4SD) and 130 women (mean age 36.5 y ± 14.4). Socio-demographic and lifestyle data are shown in Table 1. More than half the subjects were single, and they had lived in Madrid for an average of 8.5 years. The rate of obesity (based on BMI computed from reported weight and height) was twice as high in women and in men. Regular practice of exercise or sports was significantly more frequent in men than in women, which may have some bearing on the gender difference in obesity. It is also seen in Table 1 that women reported poorer health in general and as related to cardiovascular disease, which is consistent with a usually higher level of health consciousness among women than men. Smoking was uncommon among men and women. Additional information may be found elsewhere [17]. There was no age or sex difference between the participants (71% response rate) and non-participants.

**Table 1:** Characteristics of study subjects (%)

	**Men** **N = 83**	**Women** **N = 130**
Age category (years)		
< 30	45.8	35.4
30–44	38.6	41.5
≥ 45	15.0	23.1
Years in Spain		
< 6	42.2	36.9
6–10	26.5	39.2
≥ 11	31.3	23.8
Body mass index*		
< 30	89.0	76.8
≥ 30	11.0	23.2
Self-reported health**		
Very good	30.0	13.8
Good	46.3	47.2
Fair/poor	18.2	39.0
Self-reported CVD condition*		
Yes	16.9	23.1
No	83.1	76.9
Smoking status		
Non-smoker	90.4	86.9
Former smoker	4.8	6.9
Smoker	4.8	6.2
Regular practice of exercise/sports**		
No	39.0	65.9
Yes	61.0	34.1
Frequency of intake of traditional foods (per week)		
≤ Once	37.3	32.3
2–4 times	41.0	44.6
≥ 5 times	21.7	23.1

* p < 0.05 **p < 0.01 Chi^2^

### Food consumption

The average daily intake of 19 food groups by men and women is given in Table 2. Men had a significantly higher consumption of eggs, total and processed meat, fruits, cereal, bread, alcoholic beverages and animal fat. Their energy intake was also higher than that of women by about 300 kcalories (2546 ± 630 kcal in men, 2228 ± 455 kcal in women; p < 0.001).

**Table 2:** Food group consumption in Bubi men and women (grams per day)

**Food group**	**All** **N = 213**	**Men** **N = 83**	**Women** **N = 130**
Milk	427.5 ± 219.0	425.8 ± 225.1	428.5 ± 215.9
Eggs	21.0 ± 20.8	25.8 ± 3.3*	17.9 ± 11.1
Meat total	150.5 ± 60.6	165.5 ± 65.6**	140.8 ± 55.4
White meat	46.7 ± 26.8	51.1 ± 31.1	43.9 ± 23.3
Red meat	65.2 ± 40.0	69.0 ± 45.8	62.7 ± 35.8
Processed meat	38.6 ± 28.9	45.4 ± 29.4**	34.2 ± 27.8
Fish	85.6 ± 43.8	84.3 ± 40.9	86.5 ± 45.6
Vegetables	258.9 ± 95.8	251.4 ± 95.5	263.7 ± 96.0
Fruit	254.3 ± 152.4	279.7 ± 155.9*	238.1 ± 148.5
Legumes	43.7 ± 31.4	44.0 ± 31.7	43.5 ± 31.3
Cereal & pasta	152.7 ± 81.2	167.0 ± 82.7*	143.5 ± 79.2
Bread	59.6 ± 38.0	61.0 ± 35.9***	51.6 ± 37.7
Potatoes	51.6 ± 40.5	54.9 ± 42.8	49.5 ± 38.9
Nuts	5.3 ± 9.6	5,3 ± 7.7	5,4 ± 10.7
Sweets	67.0 ± 54.4	71.4 ± 40.4	52.0 ± 34.4
Sweetened drinks	302.4 ± 229.7	312.9 ± 251.9	295,7 ± 215.0
Alcoholic beverages	87.4 ± 108.4	111,8 ± 123.0*	71.8 ± 96.2
Animal fats	1.0 ± 1.9	1.4 ± 2.2*	0.7 ± 1.6
Vegetable oils and fats	17.9 ± 9.4	21.9 ± 11.0	19.6 ± 9.6
Mixed dishes	66.6 ± 53.7	63.8 ± 52.4	64.0 ± 54.2

Mean ± standard deviation (SD)

* p < 0.05 ** p < 0.01 *** p < 0.001 *t*-test

### Dietary patterns

A two-cluster solution was retained, after dropping two subjects (two women) who were in a third cluster. Food group consumption (per 1000 kcalories) is given for each cluster in Table 3. The food patterns are well differentiated. The first cluster was named 'Western' because of a significantly higher intake of sweet foods and drinks, animal fat and mixed dishes (this food group includes composite dishes such as "croquettes", soups and sauces), whereas in the'Healthier' cluster, consumption of milk and dairy products, fish, vegetables, fruits, legumes and bread were significantly higher. Two-thirds of the subjects were in the 'Western' cluster (62.5% of the men and 72.3% of the men).

**Table 3:** Food group intakes according to dietary pattern (grams/1000 kcal)

	**'Western' dietary pattern (N = 60♂; 80♀)**	**'Healthier' dietary pattern (N = 23♂; 50♀)**
Milk	171.6 ± 88.4	213,1 ± 95.1**
Eggs	9.2 ± 7.7	7.9 ± 5.5
White meat	19.6 ± 10.8	21.7 ± 10.2
Red meat	28.0 ± 16.2	26.9 ± 15.0
Processed meat	19.0 ± 10.4***	9.1 ± 8.9
Fish	30.8 ± 14.8	50.5 ± 19.7***
Vegetable	97.1 ± 33.2	147.1 ± 47.3***
Fruit	94.4 ± 42.6	139.0 ± 73.6***
Nuts	2.2 ± 3.0	1.7 ± 2.5
Legumes	15.6 ± 10.7	27.0 ± 18.5***
Cereal & pasta	66.8 ± 35.0	62.9 ± 30.6
Potatoes	23.8 ± 15.0**	17.3 ± 14.9
Bread	22.6 ± 11.6	31.3 ± 19.1***
Sweets	25.9 ± 13.6***	17.6 ± 12.0
Sweetened drinks	141.2 ± 87.8**	104.7 ± 79.6
Alcoholic beverages	39.1 ± 41.2	29.4 ± 40.3
Animal fat	0.5 ± 0.8**	0.17 ± 0.40
Vegetable oil	8.7 ± 3.9	9.1 ± 4.6
Mixed dishes	29.7 ± 21.5**	21.4 ± 17.4

Mean ± SD

* p < 0.05 ** p < 0.01 *** p < 0.001 *t*-test

### Nutrient intakes and diet quality according to dietary pattern

Intakes of macronutrients and foods (fruits and vegetables) related to the prevention of chronic diseases are given in Table 4 for the 'Healthier' and 'Western' food patterns, along with the FAO/WHO recommendations used to compute the prevention score, and the proportion of subjects meeting these recommendations. Compared with the 'Western' cluster, the 'Healthier' dietary pattern is characterized by a significantly lower intake of total fat, saturated fat (and also monounsaturated fat), cholesterol and free sugar, as well as a significantly higher intake of fibre, fruits and vegetables. A significantly lower energy intake was also observed in the healthier cluster. Only one-third of the 'Western' cluster subjects met the saturated fat criterion, compared with two-thirds in the 'Healthy' cluster; 20% of the former reached the cholesterol recommendation, compared to 32 in the latter. Significantly higher percentages of the 'Healthier' than 'Western' pattern subjects also complied with the fruits and vegetables, and sugar recommendations. Intakes of omega-3 fatty acids and fibre were very low in both clusters. Only a small proportion complied with the maximum of 30% of total energy as fat, in both the 'Western' cluster (2%) and the 'Healthy' cluster (20%).

**Table 4:** Macronutrient intakes and compliance with WHO recommendations according to dietary patterns

	**'Western' pattern** N = 140	**'Healthier' pattern** N = 71	**FAO/WHO criteria for prevention score**	**% of subjects meeting the WHO criteria**	
				
				**'Western' pattern**	**'Healthier' pattern**
Protein g	105.0 ± 24.8	102.2 ± 24.4			
% total calories as protein	17.0 ± 2.2	19.6 ± 2.4***	≥ 10% total kcal	100	100
Total fat g	103.2 ± 24.7***	79.8 ± 20.0			
% total energy as fat	37.5 ± 4.0	34.3 ± 4.3	15–30% total kcal	2.1	19.7***
Saturated fat g	30.1 ± 8.3***	22.0 ± 7.1			
% total energy as sat. fat	10.9 ± 1.8	9.4 ± 1.9	< 10% total kcal	33.6	66.2***
Monounsaturated fat g	45.5 ± 11.7***	35.6 ± 9.1			
w-3 fatty acids (FA) g	1.8 ± 0.5(*)	1.7 ± 0.5			
% total energy as w3 FA	0.65 ± 0.12	0.74 ± 0.13***	1–2% total kcal	1.4	1.4
Cholesterol mg	471 ± 235(*)	416 ± 202	< 300 mg/d	20.0	32.4***
Sugar g	59.2 ± 31.2***	37.0 ± 18.4			
% total energy as sugar	21.5 ± 9.3***	16.1 ± 7.8	< 10% total kcal	3.6	25.4***
Fibre g	19.7 ± 6.0	21.2 ± 5.6	≥ 25 g/d	17.1	25.4
Fruit & vegetable g	473 ± 178	596 ± 219***	≥ 400 g/d	54.3	80.3***
Kcalories	2484 ± 550***	2090 ± 440			

FAO/WHO criteria for 8 components of the Prevention score

* p < 0.05 ** p < 0.01 *** p < 0.001 *t*-test (*) < 0.15 p < 0.10

The micronutrient density of the 'Healthier' pattern (Table 5) was significantly higher than that of the 'Western' pattern for 9 out of 10 micronutrients; only thiamine density was similar. The percentage of subjects meeting the recommended micronutrient intakes was below 67% for calcium, iron and folate in both dietary clusters. A higher rate of folate adequacy in the 'Healthier' pattern (44% vs 22%), and a higher rate of thiamine adequacy in the 'Western' cluster were the only significant differences between clusters in the proportion of subjects meeting the recommended intakes. However, there was no difference between clusters in the overall micronutrient adequacy score (see Table 6).

**Table 5:** Micronutrient density (nutrient/1000 kcal) and total intake adequacy according to subjects' dietary patterns

	**Micronutrient density of diet (per 1000 kcal)**		**Criteria for intake adequacy (FAO/WHO)**	**% of subjects with adequate intake**	
			
	**'Western' pattern**	**'Healthier' Pattern**		**'Western' pattern**	**'Healthier' pattern**
Calcium (mg)	390	481***	1000 for ♂, 1000 for ♀ of 19–50 y, 1300 for ♀ ≥ 51 y	35	45.1
Iron (mg)	9.3	10.3***	11 for ♂, 24 for ♀ 19–50 y, 9 for ♀ ≥ 51 y	63.6	63.4
Thiamine (mg)	0.66	0.68	1.2 for ♂, 1.1 for ♀	90*	78.9
Riboflavin (mg)	0.94	1.08***	1.3 for ♂, 1.1 for ♀	97.1	98.6
Niacin (mg)	10.3	12.1***	16 for ♂, 14 for ♀	97.1	94.4
Folate (FE)	137	180***	400	22.1	43.7**
Vitamin B12 (μg)	4.9	6.1*	2.4	99.3	100
Vitamin A (RE)	713	889*	600 for ♂, 500 for ♀	95.7	98.6
Vitamin C (mg)	52	70***	45	97.1	100
Vitamin E (ATE)	5.5	5.9**	7.5 for ♀; 10 for ♂	94.3	90.1

FE = folate equivalents RE = Retinol equivalents ATE = Alpha-tocopherol equivalents

* p < 0.05 ** p < 0.01 *** p < 0.001 *t*-test

**Table 6:** Diet quality indices according to dietary clusters

	**'Western' pattern**	**'Healthier' pattern**
**Alternative Healthy Eating Index (max 87.5)**	43.7 ± 6.4	46.7 ± 6.0**
**Alternative Mediterranean Diet Score (max 9)**	4.1 ± 1.9	4.2 ± 1.7
**Prevention score (max 8)**	4.6 ± 1.2	5.7 ± 1.2***
**Micronutrient adequacy score (max 10)**	7.9 ± 1.4	8.1 ± 1.35

* p < 0.05 ** p < 0.01 *** p < 0.001 *t*-test

Among the four diet quality indexes tested, both the AHEI and our preventive score were significantly higher in the 'Healthier' pattern cluster; the AMDS and our micronutrient score were not (Table 6). The quality indexes were all significantly correlated, except for our preventive and micronutrient scores as these are purposely designed to capture different aspects of dietary quality. Our preventive score and the AMDS showed a similar correlation with the AHEI (r = 0.44 and 0.43, respectively, p < 0.01). The highest correlation was between our micronutrient score and the AMDS (r = 0.63, p < 0.01), the two indexes exhibiting no difference between dietary patterns as shown in Table 6.

### Socio-demographic and health profile according to dietary pattern

Age 30 years and above, and a longer duration of residence in Spain (≥ 11 years vs < 6 years) were significantly and independently associated with higher odds of having a 'Healthier' dietary pattern, according to logistic regression (Table 7). Women and ex-smokers also tended to aggregate in the 'Healthier' pattern group (p < 0.20). However, obesity status, physical exercise patterns, the frequency of consumption of traditional foods, and reported health or a CVD condition (diabetes, hypertension, cardiac disease or high cholesterol) showed no significant association with the dietary pattern identified with cluster analysis.

**Table 7:** Demographic and health-related factors associated with the healthier eating pattern (logistic regression)

	**'Western' pattern**	**'Healthier' pattern**	**OR (95%CI)**	**p**
**Sex %**				
Men	42.9	32.4	1.00	
Women	57.1	67.6	1.66 (0.79–3.5)	0.18
**Age %**				
< 30 y	52,1%	12,7%	1.00	
30–44 y	34,3%	53,5%	5.96 (2.38–14.9)	< .001
45 y+	13,6%	33,8%	6.34 (1.99–17.37)	< .001
**Years in Spain %**				
< 6 y	43.6%	28.2%	1.00	
6–10	39.3%	25.4%	0.76 (0.32–1.82)	0.54
11+	17.1%	46.5%	2.76 (1.16–6.57)	0.02
**Smoking status %**				
Non-smoker	92.1	80.3	1.00	
Ex-smoker	4.3	9.9	2.69 (0.71–10.27)	0.15
Current smoker	3.6	9.9	2.08 (0.42–10.36)	0.37
**Regular physical exercise %**				
No	44.9	43.7	1.00	
Yes	55.1	56.3	1.1 (0.54–2.23)	0.79
**Consumption of traditional foods %**				
≤ once/wk	35.7	31.0	1.00	
2–4 times/wk	43.6	42.3	1.0 (0.44–2.26)	1.0
≥ 5 times/wk	20.7	26.8	1.03 (.40–2.67)	.95
**BMI status %**				
Normal	84.4	76.1	1.00	
Obese (BMI > 30)	15.6	23.9	0.85 (0.36–2.05)	0.72
**Self-reported health %**				
Very good	22.6	16.2	1.00	
Good	48.9	41.2	0.74 (0.28–1.95)	0.54
Fair/poor	28.6	42.6	1.21 (0.43–3.44)	0.72
Self-reported CVD conditions %*				
No	85.7%	66.2%	1.00	
Yes	14.3%	33.8%	1.51 (0.56–3.48)	0.48

*Self-reported diabetes, hypertension, cardiac disease, high cholesterol

## Discussion

### Dietary patterns and quality in the Bubi population

In the adult Bubi population of Madrid, we identified two dietary patterns using cluster analysis. The dietary phenotype called 'Healthier' was indeed healthier than the 'Western' type as confirmed by two dietary quality indexes, the AHEI and our prevention score. This illustrates the usefulness of such indexes to nutritionally appraise eating patterns. The second cluster (~70% of subjects) was termed 'Western' which usually refers to a dietary pattern emphasizing energy-dense, fat-rich foods [33], but a label of 'unhealthy', as used in Mauritius might also be appropriate [34]. In societies undergoing 'westernisation', meat, potatoes, white bread, fast food and dairy products are considered typical features of the Western eating pattern, but so are whole-wheat bread, dairy products, salads and soft drinks in certain cases [34]. In our study, the "Healthier" dietary pattern was significantly better than the 'Western' pattern for almost all components of the "prevention" and "micronutrient adequacy" scores that we designed previously [28-30]. Overall, Bubi diets may be somewhat protective because mean consumption of fruits and vegetables is above the WHO recommended 400 g per day and mean level of saturated fat is around the recommended maximum of 10% (and because of a liberal intake of monounsaturated fat) in both dietary pattern groups, although the percentage of energy from omega-3 fatty acids was lower than the guidelines. More than the total amount of fat, the ratio of monounsaturated to saturated fatty acids should be of interest since monounsaturated fat is reportedly preventive of CVD and diabetes [35]. This ratio was well above 1 in both the 'Healthy' and the 'Western' food clusters (1.7 ± 0.4 vs 1.5 ± 0.3; p = .005). Notwithstanding, the proportions of subjects not meeting the guidelines are well above 50% not only for fat (except saturated fat in the 'Healthy' cluster), but also for cholesterol, sugar and fibre, in both clusters. Furthermore, with respect to micronutrients, less than two-thirds of subjects reached the recommended intakes for calcium, iron and folate in both clusters. Therefore, it appears that dietary quality is wanting in the Bubi population. Yet, comparison with data collected in the 90s in Madrid [17] would suggest a somewhat more preventive diet in the Bubis, who derive a lower percentage of energy from total, saturated and monounsaturated fat, while the percentage contribution of carbohydrate and protein is slightly higher.

There are not many reports on quantitative intakes of immigrants to compare the results of the present study with. In Haiti immigrants in Montreal (Canada), we observed a much higher proportion of compliance with the WHO fat, cholesterol and sugar guidelines than in the Bubis, but a much lower rate of compliance with the fibre, and fruit and vegetable guidelines, in the 'Western' dietary pattern as well as in the 'Traditional' pattern (the healthier cluster) [27]. Regarding micronutrient adequacy, calcium appeared much higher, vitamin B12 and vitamin A somewhat higher and folate much lower in the Bubi compared with the Haiti immigrants.

### Socio-demographic and health profile of subjects with healthier eating patterns

A higher likelihood of having a healthier diet was observed in subjects aged 30 years and above, as well as among subjects who had lived longer in Spain (11 years or more, compared with less than 6 years). Considering that the effects of age and time of residence in Spain were independent, this suggests that younger people and more recent immigrants are early adopters of the 'Western' dietary pattern. In several other food patterning studies, a western type of diet was indeed found to be more highly prevalent among younger people, including in Mauritius [34]. Among the Haiti immigrants in Montreal, we also observed that subjects with a higher quality diet had immigrated earlier than those with a 'western' type diet, who were more recent immigrants, and younger [29]. In Australia, it was observed that Greek immigrants retained part of their traditional (and protective) foods and even returned to the traditional Greek food pattern with advancing years, which is part of the explanation for continued lower mortality in first generation immigrants compared to Australians [10,36].

In the present study, obesity was not significantly associated with eating patterns in the multivariate model, nor was a reported CVD condition. In other cross-sectional [37] or longitudinal [38] studies, dietary quality scores were not consistently correlated with biomarkers of diet-related chronic diseases. We did not find either an association of food pattern or dietary quality scores (micronutrient adequacy and preventive scores as used in the present study) with metabolic factors of cardiovascular disease risk in cross-sectional studies among Haitians living in Montreal [29]. According to one review, the protective effect of healthy dietary patterns is usually modest [5]. However, in a recent meta-analysis comprising more than 1.5 million healthy subjects, greater adherence to a Mediterranean diet was associated with a reduced risk of overall mortality, cardiovascular mortality, cancer incidence and mortality, and incidence of Parkinson's and Alzheimer's disease [31]. Large samples, or else longitudinal studies, may better exhibit the association of diet quality indexes with socio-demographic factors and health behaviors on the one hand, and with chronic disease risk factors on the other hand. Interestingly, a recent report on the INTERHEART Study, a large case-control study on acute myocardial infarction (AMI) in 52 countries, showed an association between biomarkers of AMI and dietary patterns [39]. Three dietary patterns were identified using factor analysis on the basis of a qualitative FFQ comprised of 19 food items: "Oriental", "Western" and "Prudent". The authors observed a clear inverse association of AMI with the prudent diet, a U-shaped association with "Western" diet levels, and no association with the "Oriental" dietary pattern. Furthermore, a dietary risk score based on only seven food items was strongly associated with AMI risk. This important study revealed that similar eating patterns are found in different parts of the world and underlines the adverse impact of globalization on dietary patterns, with an estimated 30% of AMI attributable to unhealthy diets [40]. Aside from large cross-sectional studies of this sort, longitudinal studies among subjects without a history of CVD, diabetes or hypertension help clarify the association between diet quality and metabolic risk factors for chronic disease, as in the Whitehall II study [41]. Four dietary pattern clusters were identified at baseline: 'Unhealthy', 'Sweet', 'Mediterranean-like' and 'Healthy'. The 'Healthy' pattern reduced the risk of coronary death or non-fatal infarction and diabetes. These studies provide some evidence for the universal nature of dietary factors of cardio-metabolic disease risk and show the relevance of developing a limited number of dietary quality indexes for international use, although it has been suggested that "one size [of dietary recommendations] does not fit all", and that 'protective' diets may not be equally protective for all race-ethnicity groups [42]. The ever increasing variety of such indices [43] may not be warranted.

### Relationships among dietary quality indexes

The advantage of combining normative, or *a priori*, dietary scores with data-driven scores is that they are based on existing knowledge of optimal dietary intakes and provide a clear nutritional benchmark [44]. The higher quality of the 'Healthier' dietary pattern in Bubi subjects was reflected in two out of the four scores tested, the AHEI and our prevention score based on FAO/WHO criteria. The AMDS was not different between clusters most likely because overall consumption of food groups typical of the Mediterranean diet was relatively high in this immigrant group, possibly owing to the influence of the host dietary culture. Indeed, consumption of fruits and vegetables, cereals and legumes was on the high side when comparing our data with that of a cohort study conducted in Spain [35]. As mentioned earlier, the ratio of monounsaturated: saturated fatty acids was high in both dietary pattern clusters, which is typical of a high intake of olive oil. Interestingly, the AMDS is the score that showed the highest correlation with our micronutrient score (r = 0.67), which is ascribable to the prominence given in the AMDS to major food sources of micronutrients (vegetables, fruits, legumes, fish). The AMDS was significantly correlated with our preventive score, although loosely (r = 0.13 p < 0.001), whereas both the AMDS and our preventive score were equally correlated with the AHEI (r > 0.4). There is not at the present time any single index of dietary quality that can be considered inclusive and applicable anywhere. As underlined before [2], dietary pattern clustering requires careful judgment. We support that using dietary quality indexes may help the investigator make a final decision, but the dilemma is to decide how many and which indexes. Using only one such index may be misleading. In the present study, for instance, had we used only the Mediterranean diet score, the higher quality of the 'Healthier' dietary pattern would not have been confirmed since the score was not different from the 'Western' pattern.

### Limitations of the study

One limitation of the present study is the lack of data on socio-economic status and on biomarkers of CVD risk other than self-reported weight and height and self-assessed health condition, let alone the small size and cross-sectional nature of the study. Another limitation may be the use of a FFQ for immigrants. As suggested [2], FFQ may occult differences between ethnic groups, when specific and typical foods are collapsed into larger groups (for instance, cassava with other tubers, tortillas with bread, etc.). Despite the fact that a pilot study resulted in the addition of six traditional food items consumed by the Bubi people in Spain to the FFQ validated in Spanish population groups, it is possible that the FFQ did not allow the identification of a traditional food cluster, and therefore stages in dietary transition or acculturation, which we could do in Haitians of Montreal with food intakes based on three non-consecutive 24-hour recalls [29].

## Conclusion

Cluster analysis, preferably in combination with dietary quality assessment, may be particularly well suited to the study of food patterns among immigrants, if possible in connexion with the duration of the residence in the host country and other socio-demographic parameters, in order to identify population segments that may be at higher risk because of their diet. In the present study, younger subjects and more recent immigrants may be at higher risk because of a poorer diet, and should be priority targets for nutrition communication. It is hoped that the present trend towards proliferation of dietary quality indexes will be reversed so that a limited number of such indexes will be standardized and validated for international use.

## Abbreviations used

AHEI: Alternative Healthy Eating Index; AMI: Acute Myocardial Infarction; AMDS: Alternative Mediterranean Diet Score; CVD: Cardiovascular Disease; DQI-I: Diet Quality Index International; FFQ: Food Frequency Questionnaire; FAO: Food and Agriculture Organization; SD: Standard Deviation: WHO: World Health Organization.

## Competing interests

The authors declare that they have no competing interests.

## Authors' contributions

HD designed and conducted the statistical analyses, and drafted the paper with the assistance of JV. AG was responsible for collecting the data under the supervision of JV; she also contributed her comments and additional information for the paper.
